# Supplementary material for: Targeting FGFRs Using PD173074 as a Novel Therapeutic Strategy in Cholangiocarcinoma
Source: Cancers (Basel). 2023 Apr 28;15(9):2528. doi: 10.3390/cancers15092528 (PMC10177182; doi:10.3390/cancers15092528)
Supplement: Supplementary file 1 [file cancers-15-02528-s001.zip › Supplementary Figures.pdf]

S1

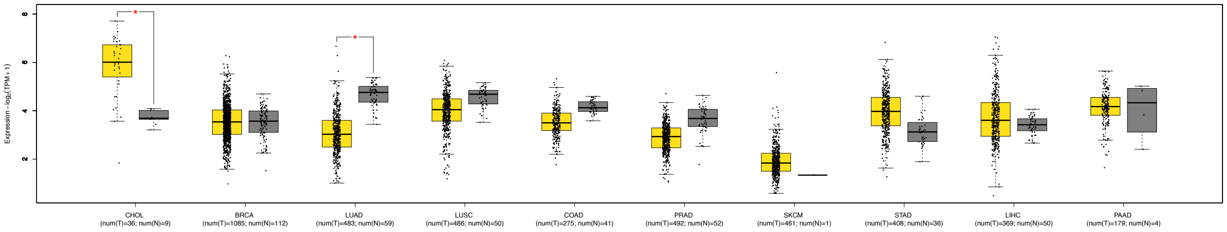

Figure S1. Box plot shows the mRNA expression level og FGFR1-4 in TCGA database for major cancer types. Graphs were plotted using online tool, GEPIA2 (http://gepia2.cancer-pku.cn).

CHOL = cholangiocarcinoma
BRCA = breast cancer
LUAD = Lung adenocarcinoma
LUSC = Lung squamous cell carcinoma
COAD = Colon adenocarcinoma
PRAD = Prostate adenocarcinoma
SKCM = Skin Cutaneous Melanoma
STAD = Stomach adenocarcinoma
LIHC = Liver hepatocellular carcinoma
PAAD = Pancreatic adenocarcinoma

S2

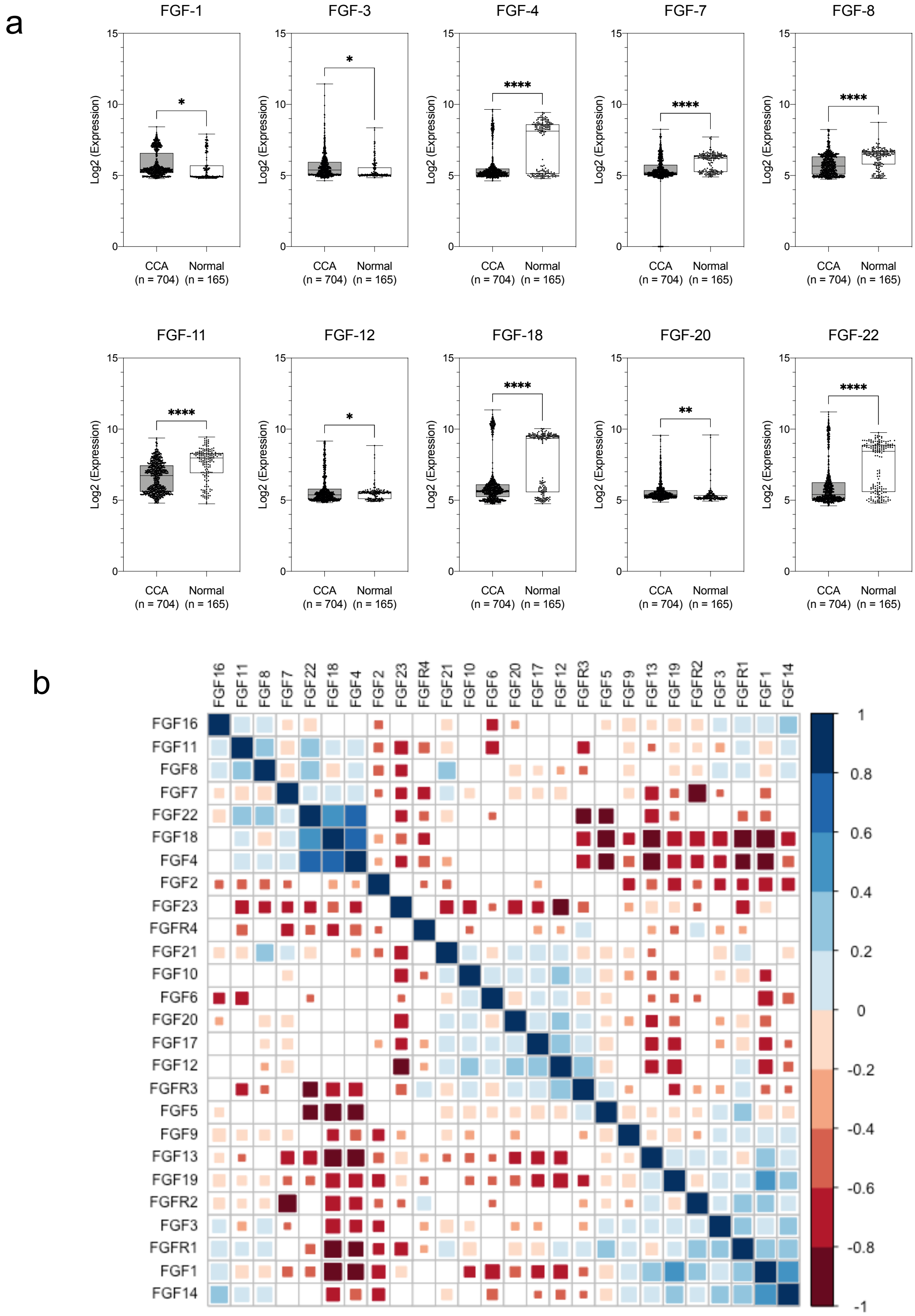

Figure S2: Expression and correlation of FGF ligands and receptor family in CCA

A) Boxplot histograms represent the comparative gene expression of FGF-1, FGF-3, FGF-4, FGF-7, FGF-8, FGF-11, FGF-12, FGF-18, FGF-20 and FGF-22.

B) A correlation matrix of FGF ligands and receptors subfamilies. Pearson correlation coefficients (r) were calculated for pairs and plotted using the *corrplot* package in R. The matrix was reordered based on hierarchical clustering using the complete linkage method. Stronger correlations are represented by darker colours and larger squares. Significant enrichments are depicted in red and depletions in blue. Colour indicates correlation (blue, positive correlation (r = +1); red, negative correlation (r = -1). The legend on the right side of the correlogram shows the Pearson correlation coefficients with their corresponding colours.

S3

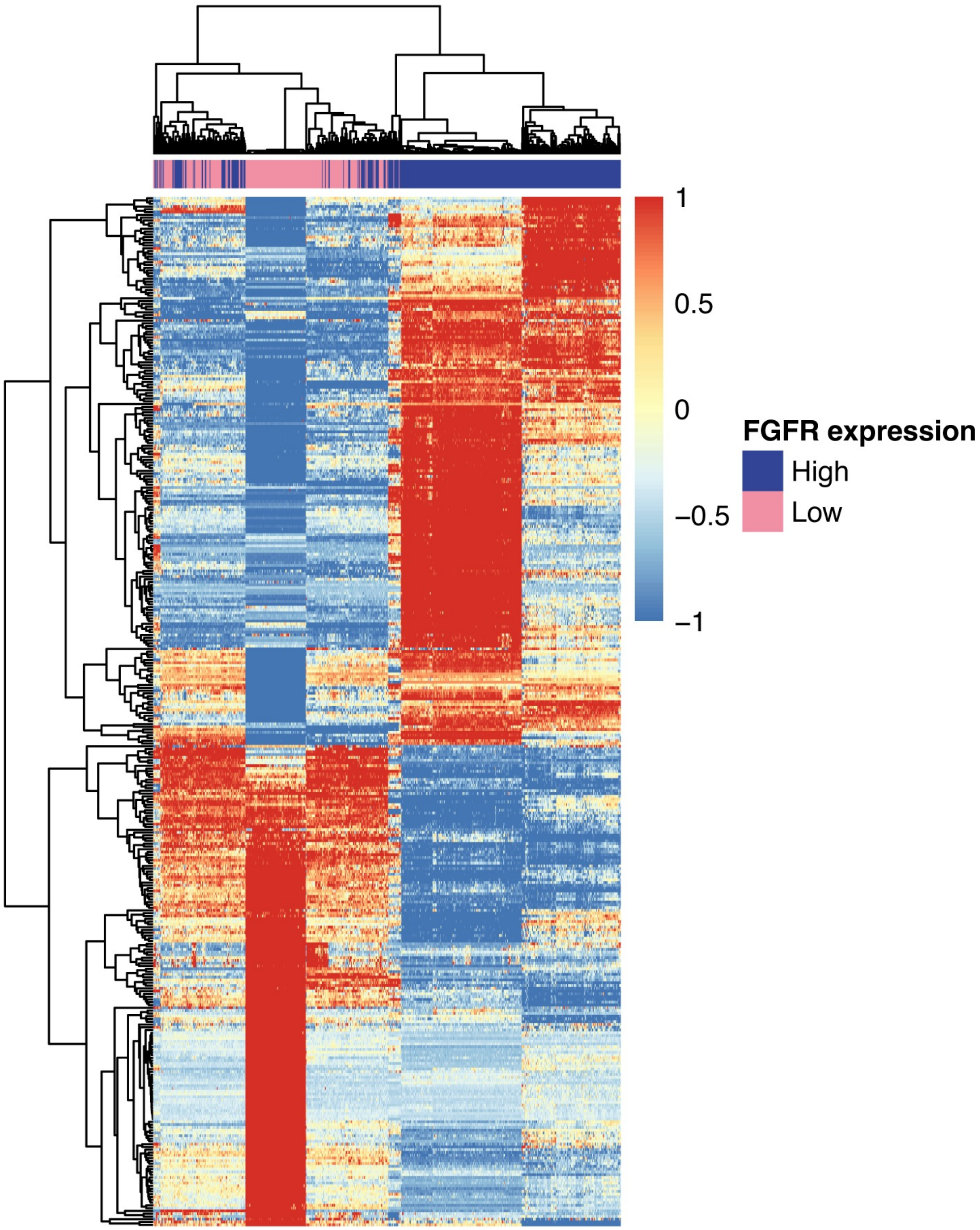

Figure S3. Heatmap depicts the differentially expression genes (DEGs) from comparing high and low FGFR expression groups. Heatmap was constructed using pheatmap package using R. 4.0

S4

M213

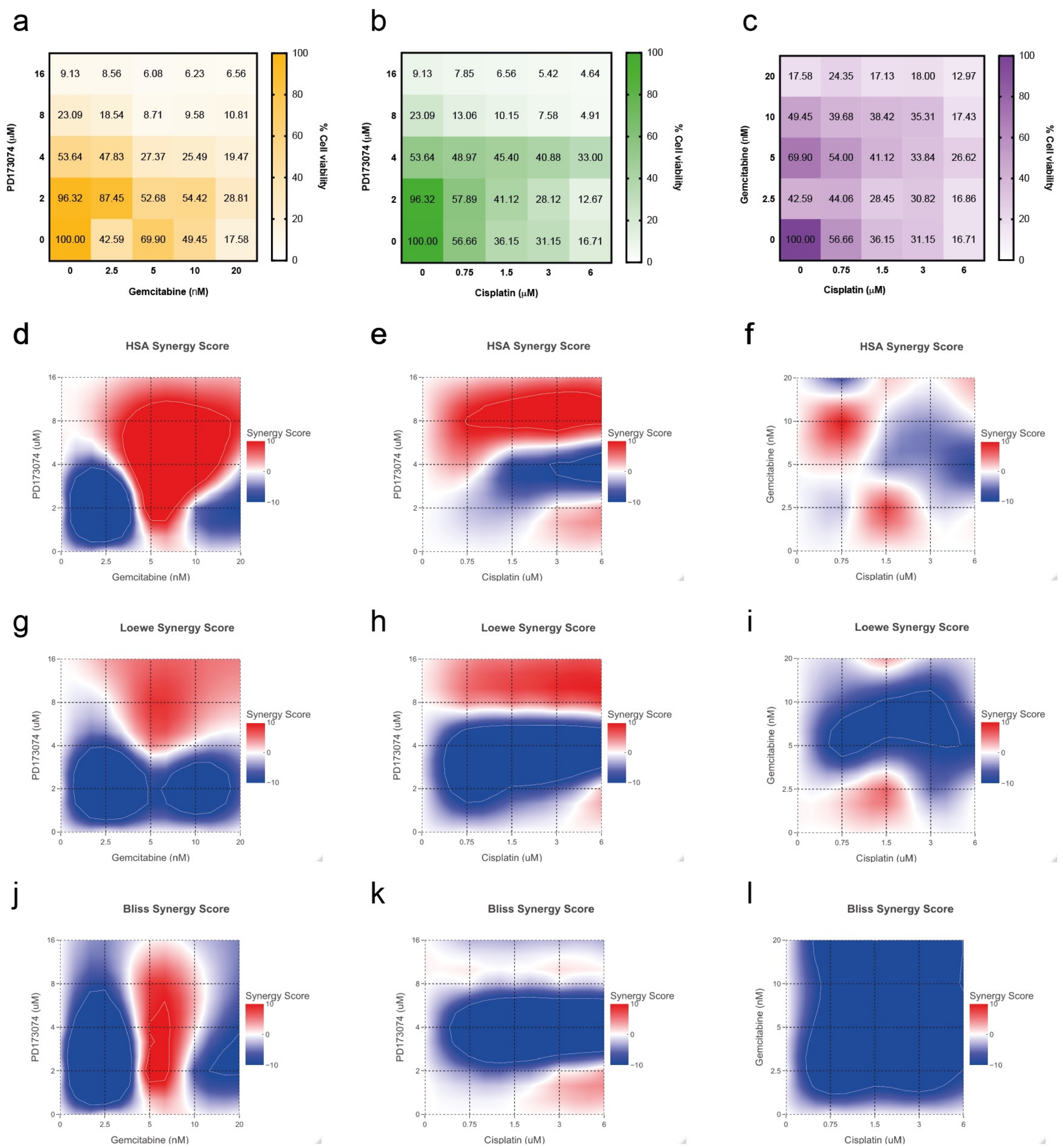

Figure S4. Combination treatment with PD173074 and erlotinib is synergistic in CCA cell line, KKU-213. The dose-response matrix shows percentage cell survival in combination amongst. A) PD173074 and Gemcitabine, B) PD173074 and Cisplatin and C) Gemcitabine and Cisplatin. Synergy scores were calculated according to d,e,f) HSAmodel, g,h,I)Loewe's additivity mathematical model and j,k,l) Bliss Independence model using the R package SynergyFinder.

S5

RBE

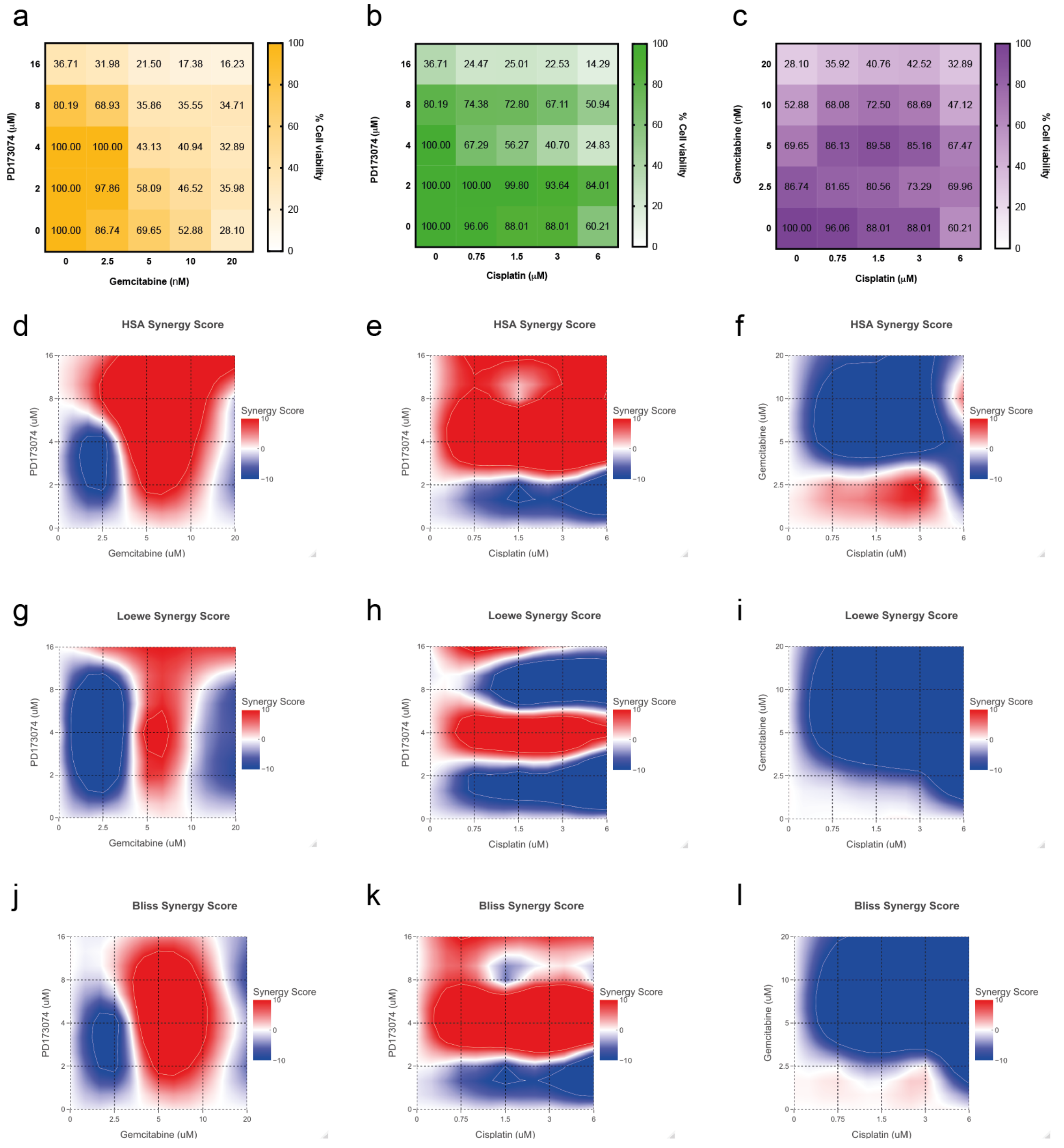

Figure S4. Combination treatment with PD173074 and erlotinib is synergistic in CCA cell line, RBE. The dose-response matrix shows percentage cell survival in combination amongst. A) PD173074 and Gemcitabine, B) PD173074 and Cisplatin and C) Gemcitabine and Cisplatin. Synergy scores were calculated according to d,e,f) HSAmodel, g,h,I)Loewe's additivity mathematical model and j,k,l) Bliss Independence model using the R package SynergyFinder.

S6

a

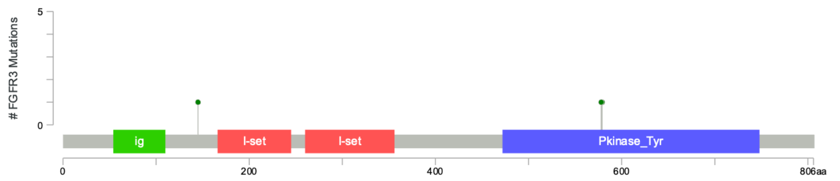

b

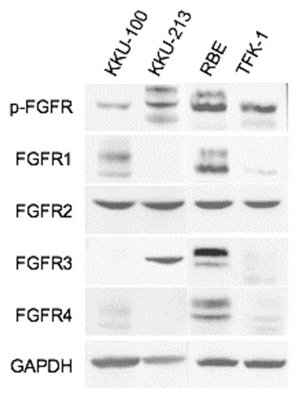

Figure S6. Mutation and Protein expression of FGFRs in CCA cell lines

A) Mutation plot showing the two different FGFR mutations present in CCA cell lines in this investigation. No other mutations or fusions in FGFR were observed in these cell lines.

B) Base-line FGFR expression in different cell lines investigated.

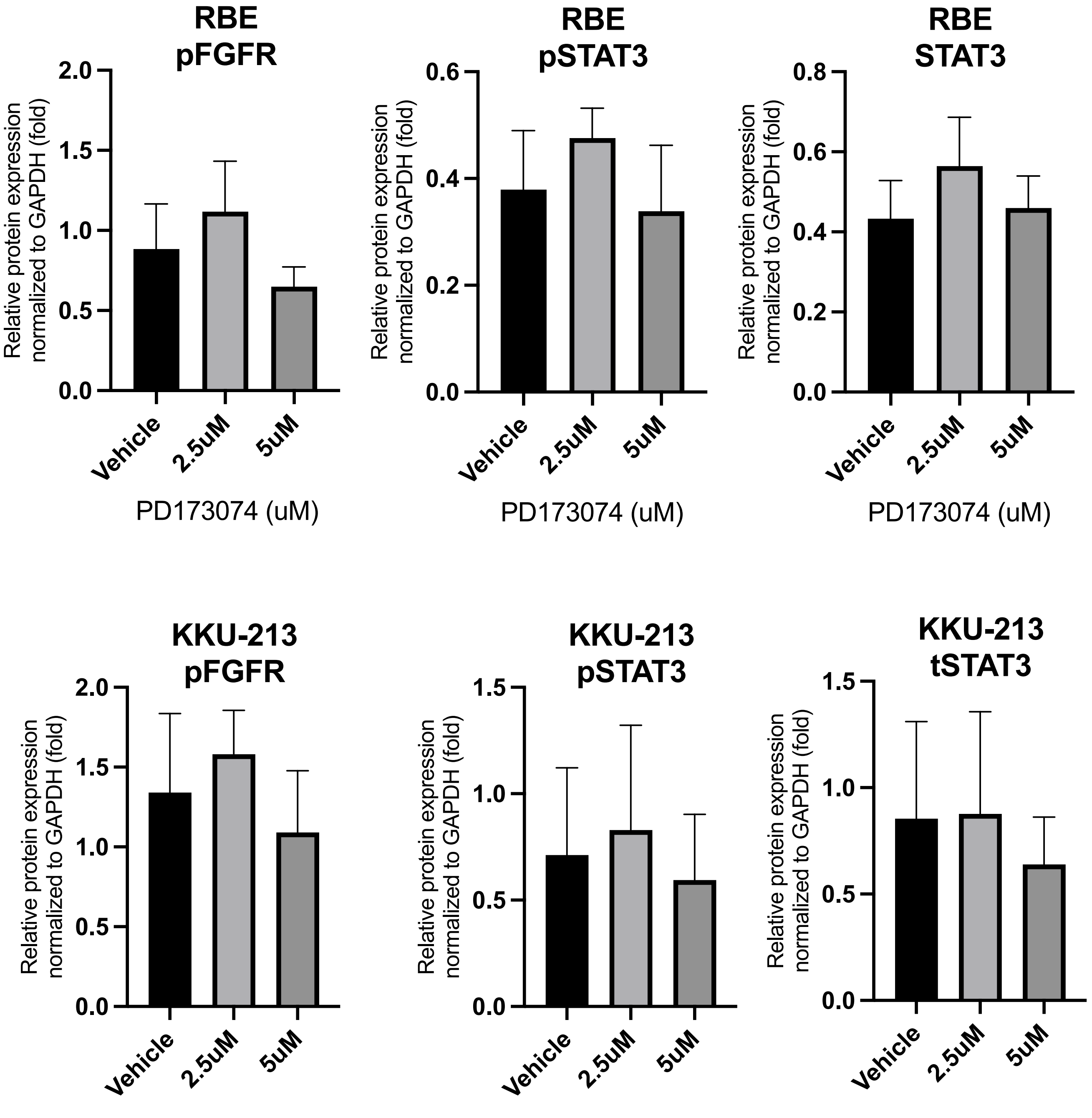

Figure S7. Relative protein expression of p-FGFR, p-STAT3 and STAT to GAPDH in RBE and KKU-213 cells following 24h treatment with PD1730374
